# Transcultural adaptation and theoretical models validation of the Spanish version of the Self-Care of Diabetes Inventory

**DOI:** 10.3389/fmed.2024.1423948

**Published:** 2024-09-10

**Authors:** Jesús Martínez-Tofé, Davide Ausili, Nelia Soto-Ruiz, Iván Santolalla-Arnedo, Angela Durante, Marco di Nitto, Yuliia Lysanets, Regina Ruiz de Viñaspre-Hernández, Clara Isabel Tejada-Garrido, Mercedes Sánchez Barba, Vicente Gea-Caballero, Raúl Juárez-Vela

**Affiliations:** ^1^Doctoral Program in Health Sciences, Public University of Navarra, Pamplona, Spain; ^2^Faculty of Health Sciences, Research Group in Care, University of La Rioja, Logroño, Spain; ^3^Department of Medicine and Sugery, Università degli Studi di Milano-Bicocca, Milan, Italy; ^4^Department of Health Sciences, Public University of Navarra (UPNA), Pamplona, Spain; ^5^IdiSNA, Navarra Institute for Health Research, Pamplona, Spain; ^6^Sant’Anna School of Advanced Studies, Health Science Interdisciplinary Center, Pisa, Italy; ^7^Fondazione Toscana “Gabriele Monasterio”, Pisa, Italy; ^8^Department of Health Sciences, University of Genoa, Genoa, Italy; ^9^Department of Foreign Languages with Latin and Medical Terminology, Poltava State Medical University, Poltava, Ukraine; ^10^Department of Statistics, University of Salamanca, Salamanca, Spain; ^11^Faculty of Health Sciences, Research Group Community and Health SALCOM, International University of Valencia, Valencia, Spain

**Keywords:** validation, diabetes self-care inventory, diabetes mellitus, self-care, Spain

## Abstract

**Background:**

For patients with diabetes mellitus, self-care is crucial because it prevents complications and helps preserve quality of life. Clinicians and researchers require effective tools for assessing self-care behaviors across various dimensions to identify individual needs and maximize resource allocation. The aim of this study was to evaluate the validity and reliability of the Spanish version of the Self-Care of Diabetes Inventory (SCODI).

**Methods:**

Two hundred eighteen participants with DMT1 and DMT2 who were recruited through convenience sampling from a university hospital participated in our cross-sectional study. After translation and cultural adaptation, the enrolled patients answered the questions. We performed an exploratory factor analysis (EFA) on each of the SCODI scales and Confirmatory factor analysis (CFA) was performed using our models which appropriate fit indices.

**Results:**

The original structure of the four-dimensions tool was confirmed. The overall consistency across the four scales was assessed by Cronbach’s alpha: self-care maintenance (0.766), self-care monitoring (0.790), self-care management (0.771), and self-care confidence (0.936). The model fit yielded a chi-square index of 1.028 with 773 degrees of freedom. Confirmatory factor analysis showed a good fit, thereby affirming the reliability of the model.

**Conclusion:**

The internal consistency and reliability of the SCODI Spanish version are deemed adequate. This tool is appropriate when it is desired to evaluate the self-care practices of Spanish persons suffering from diabetes due to its good psychometric qualities.

## 1 Introduction

Diabetes mellitus (DM) is considered a global public health problem (1). Although it is a non-communicable pathology, it has reached the status of a pandemic disease because of its instantaneous spread (2). According to the tenth edition of the IDF Diabetes Atlas, the worldwide prevalence of diabetes was estimated to be 9.3% in 2019, with 463 million people living with the disease (3). Furthermore, the prevalence of diabetes is projected to increase to 10.2% (578 million) by 2030 and 10.9% (700 million) by 2045 (4). In Spain, the prevalence has reached 14.8% of the adult population, ranking it second highest in Europe according to IDF (3).

DM and its complications are relevant components that contribute significantly to morbidity and premature death (5). Stroke, diabetic nephropathy, neuropathy, retinopathy, cardiovascular diseases, pregnancy problems, low quality of life, heavy financial burden, and an increased chance of dying young are all linked to DM (6). As far as premature mortality due to other relevant non-communicable pathologies is reducing, deaths attributable to DM have increased by 5% (7). The great negative impact of DM on communities and on the sustainability of the different healthcare systems means that its treatment and prevention have become a shared universal priority (8).

For those with DM, it is crucial to maintain an active lifestyle, a nutritious diet, follow-up appointments, screening tests, blood glucose monitoring, and daily insulin administration when needed; these actions can be summed up as self-care (9). Given that self-care improves quality of life and metabolic control, and reduces hospitalizations, mortality rates and the risk of diabetic complications (diabetic foot, diabetic retinopathy, diabetic neuropathy and diabetic renal pathology), it is evident that it should become one of the most relevant strategies in the treatment of DM (10). According to the World Health Organization, self-care is “the ability of individuals, families and communities to promote health, prevent disease, maintain health and cope with illness and disability” (11). Jaarsma et al. (12) advise using theory to create a framework for planning and evaluation when creating self-care interventions. However, this is often not done. As an example, we can observe the lack of conceptualization and measurement of self-care in Canadian and American studies, indicating the need for further studies to define self-care accurately and develop a theoretical framework for self-care in DM. They recommended studying Riegel’s concept of self-care as a perfect basis for use in patient care and research (13).

The “process of health maintenance through health promotion and disease management practices” (14) with or without the assistance of a health professional is what is meant to be understood by self-care, according to Riegel’s “middle-range theory of self-care of chronic illness” (11). The capacity to identify individuals at risk of unfavorable outcomes is greatly aided by the availability of valid and trustworthy instruments to evaluate diabetic self-care (15). At present, there are at least 16 accessible tools for assessing self-care in DM (16). Ten tools are unidimensional, focusing only on aspects such as caloric intake or exercise (17). The remaining tools are multi-dimensional. Of these, the two most popular were created before 2005 and have not been updated with new clinical data. Of the six, only two (16, 17) employ a clear theoretical framework and do not measure diabetes self-care behaviors (18, 19). Several systematic reviews have pointed out that there is insufficient data to support a sound theoretical framework or validation procedure. Therefore, a theoretically grounded and psychometrically reliable instrument is needed to assess diabetes self-care (16, 17).

Based on the “middle-range theory of self-care of chronic illness,” Ausili et al. developed the “Self-Care Diabetes Inventory” (SCODI) in 2017. It has been clinically revised and shown to be a valid and trustworthy tool for measuring diabetics’ self-care. Multidimensional model-based reliabilities were between 0.81 (maintenance) and 0.89 (confidence). Significant associations were found between self-care maintenance and HbA1c (*p* = 0.02) and between self-care monitoring and diabetes complications (*p* = 0.04). May be useful for academics as well as medical professionals (20). Key aspects of the theory measured by the tool include maintenance, monitoring, management and confidence. Maintenance consists of behaviors aimed at preserving health, mental and physical balance, or improving well-being through exercise, nutrition, or medication. Self-care monitoring involves “listening to the body,” i.e., watching and analyzing signals and cues, including blood glucose monitoring. It is the connection between the maintenance and management of self-care. The responsive actions a person takes in response to signals and cues, including hypo- or hyperglycemia, are self-care management (e.g., adding more insulin). Confidence is a component that greatly influences self-care maintenance, monitoring, and management; however, it is not an element of self-care. It shows persistence despite difficulties and confidence in one’s own ability to practice self-care (9, 10, 20). In sets of people with T1DM and T2DM, the SCODI demonstrates the same dimensionality, with little variation in factor loadings for each component and each scale, and excellent reliability for each scale in the two sets (9).

The SCODI is in the public domain and is freely available on the Internet in 14 languages.^1^ It has been shown to be generalizable to other cultures and languages (10, 21–23). Self-care habits are a reflection of language and culture (24). Therefore, it is necessary to translate, culturally adapt, and evaluate the psychometric properties of the SCODI in various languages and regions. It is common practice to adapt a tool from one language to another. To confirm equivalence, researchers must subsequently examine the psychometric qualities (25). At present, there is no validated Spanish version of the SCODI, despite Spanish being the second most spoken language globally and the fourth in terms of the total number of speakers (26). The purpose of this work was to translate, adapt, and evaluate the psychometric properties of the Spanish version of the Self-Care Diabetes Inventory (SCODI).

## 2 Materials and methods

### 2.1 Study design

This study was a methodological and cross-sectional investigation aimed at assessing the validity and reliability of the SCODI (Self-Care of Diabetes Inventory, Monza, Italy), a Spanish translation of the Ausili et al. (20) self-care measures instrument for persons with DM.

Development of the SCODI Spanish version.

The SCODI was converted from its English to Spanish version, and its psychometric properties were then tested. We employed the methodology outlined by Beaton et al. for cultural adaptation and large-scale translation, which involved several steps: translation, synthesis, back-translation, retro-synthesis, review of the translated version by a committee of professionals, and preliminary testing (27). Throughout the entire process, the indications of the original authors for the translation process and cultural adaptation to other languages of the tool were also respected at all times (self-care-measures website). Therefore, the English version of SCODI was used as the basis for translation. Two advanced translators, with Spanish as their mother tongue, translated the SCODI freely. One was a health professional (nurse) familiar with the instrument and its properties, and the other was a linguist expert in the original language, without health knowledge. Two variants were obtained, which were combined into one Spanish version through a work session between translators and scholars. Then, two professional translators, with English as their native language, who had not previously observed the original version in English and had no knowledge of self-care, freely performed the back-translation by taking the new Spanish version of the SCODI to obtain the English version. The translation team and the researcher combined both variants into a final version in back-translated English, which was then sent to the original creator of the instrument for review of the transcription’s accuracy and acceptance. Finally, cognitive interviews were conducted with a sample of 30 people to implement minor changes in the final version.

### 2.2 Procedures and data analysis

A single-center cross-sectional study was carried out in northern Spain (Logroño, La Rioja). To confirm validity, different authors indicate that the appropriate and reliable sample size for factor analysis should be 5–10 persons per item (28). SCODI does not provide a global measure of self-care, but was designed as an inventory with four different constructs. The largest scale, with 12 items, is self-care maintenance, so a sample of 84 people would be adequate. However, to support a more stable analysis, and taking into account the original validation of SCODI and its international translations, we started with a minimum of 200 (10, 20–23). Convenience sampling was used, inviting all patients admitted between January 2022 and January 2023 who attended the multipurpose surgical ward of the Hospital Universitario San Pedro de Logroño (the section where the study was authorized). The patients selected met the following requirements: medical diagnosis of T1D1 or T2DM, age of 18 years or older, signing the informed consent form, and understanding the objectives of the analysis. Participants with a medical diagnosis of DM less than 1 year old, and those with relevant cognitive impairment, judged by receiving less than four points on a “six item screener” questionnaire (29), were rejected. Clinical and demographic information was collected from each patient using a self-report questionnaire. In addition to the SCODI, we administered a sociodemographic questionnaire to collect information on the properties and components involved with MD, as well as other forms for parallel inquiries. The whole process was performed directly by trained technical staff.

Descriptive statistics were performed for both the primary sociodemographic and clinical characteristics of the sample (frequency and percentage) and for the SCODI item and subscale scores (mean, standard deviation, skewness, and kurtosis).

Subsequently, we performed exploratory factor analysis (EFA) in order to determine the number of latent constructs and the underlying factor structure of each SCODI scale.

For the EFA, we used principal axis factoring and ProMax oblique rotation. Data analysis was performed using Jamovi and IBM SPP-AMOS V24 (IBM Corporation, New Orchard Road Armonk, New York, NY, USA) (30–42).

### 2.3 Diabetes self-care inventory

All candidates completed all 40 items of the Spanish version of the SCODI, which was scored on a 5-point Likert scale. Since the SCODI is essentially a scale comprising four subscales that independently measure the four fundamental items of diabetes self-care according to Riegel’s middle-range theory (maintenance, monitoring, management, and confidence in self-care), it does not provide a single final score. According to the authors’ indications, we have to perform the calculation of standardized scores for each of the four subscales.^2^ Each category receives a score ranging from 0 to 100, where higher scores correspond to better self-care. A threshold score of 70 for each measure has been used in prior research to distinguish between adequate and insufficient self-care (43). Using standardized scales allows comparison of different areas of self-care, identifying problems and associations.

### 2.4 Ethical considerations

The local research ethics committee of La Rioja approved the project (reference number CEImLAR P.I. 572). Before using the study instruments, each participant completed an informed consent form after being fully informed of the objectives of the analysis and of their ability to withdraw at any time. Cooperation was voluntary and the alphanumeric coding of each participant’s form with the data maintained anonymity and confidentiality throughout the study period. The same anonymous code identifies each individual in our database. The working protocol complied with the principles of the Declaration of Helsinki.

## 3 Results

A total of 218 people agreed to participate in the study by fulfilling the requirements. The sample’s average age was 54 years, with an equal distribution of males and females. The majority were married, had kids, lived with a spouse, and had either a primary or university education. All patients were diabetic, with a prevalence of hypertension in 45%, followed by peripheral vascular pathology (21%), congestive heart disease (20%), anemia (15.2%), and sleep apnea (14.3%). The majority seek diabetes assistance through health centers and outpatient facilities (Table 1).

**Table 1:** Lists the sample’s primary sociodemographic and clinical attributes (*n* = 218).

Variables	*n*	%
Sex		
Female	108	49.5
Male	110	50.5
Marital status		
Single/unmarried	62	28.4
Married	120	55.0
Separated/divorced	18	8.3
Widowed	18	8.3
Education		
Elementary	56	25.7
Secondary school	18	8.3
Diploma	41	18.8
Bachelor’s degree	26	11.9
University	77	35.3
Current occupation		
No active	121	55.8
Active	96	44.2
Coexistence		
Alone	33	15.2
Accompanied	184	84.8
Children		
No	75	34.6
Yes	142	65.4
Economic status		
Have more than enough to sustain life comfortably	48	22
Have sufficient means to support oneself	154	70.7
Lack of adequate resources to meet basic needs, facing ongoing hardship	16	7.3
Smoker		
Yes	28	12.9
No	190	87.1
Drink alcohol		
Yes	59	27
No	159	73
Diabetes-related hospitalizations		
Yes	20	9.3
No	196	90.7
Body mass index		
<18.5	5	2.3
18.5–24.99	96	45.1
25–29.99	73	34.3
≥30	39	18.4
Type of diabetes		
DM1	107	49
DM2	111	51
HbA1c		
5–6.9%	124	56.9%
7–7.9%	71	32.5%
8–15.9%	23	10.6%
Charlson comorbidity index score		
Mild	95	43.8
Moderate	37	17.1
Severe	86	39
Diabetes progression		
Managed through diet alone	3	1.4
Without end-organ damage	200	91.8
With end-organ damage	15	6.8
Diabetes support services:		
None		
No	10	4.6
Yes	208	95.4
Health center visits		
No	68	31.2
Yes	150	68.8
Daily hospital visits		
No	212	97.2
Yes	6	2.8
Outpatient facilities		
No	112	51.4
Yes	106	48.6
Telephone support		
No	209	95.9
Yes	9	0.5
Telecare		
No	217	99.5
Yes	1	0.5
Others (associations, support groups …)		
No	209	95.9
Yes	9	4.1

Table 2 shows the descriptive analysis of the 40 items of the Spanish version of the SCODI for the sample of 218 persons with DM. In the first scale (items 1–12; self-care maintenance) the question “Do you take all your medications?”(SCODI12) obtained the highest score, while the question “Do you dedicate time to physical exercise?”(SCODI2) obtained the lowest score. On the second scale (items 13–20; self-care monitoring) the question “Do you regularly monitor your blood sucrose levels?” (SCODI 13) scored the highest, compared with the question “Do you monitor your blood pressure?”(SCODI 15), which scored the lowest. “Do you measure your blood sucrose level once you feel symptoms?”(SCODI21) received the highest score on the third scale (items 21–29; self-care management), and “Do you ask a family member or friend for advice?” was the item with the lowest score (SCODI23). Finally, the question with the highest score on the fourth scale (items 30–40; confidence in self-care) was “Do you take your medications correctly?” (SCODI32), and the question “Do you comply with nutritional and physical activity advice?” (SCODI 31) received the lowest score.

**Table 2:** Descriptive factors data for the items included in the SCODI Spanish version.

Item	Mean	SD	Skewness	Kurtosis
SCODI1	3.67	1.29	−0.61	−0.78
SCODI2	2.84	1.66	0.14	−1.61
SCODI3	4.08	0.95	−1.01	0.63
SCODI4	3.39	1.18	−0.30	−0.73
SCODI5	4.30	1.19	−1.69	1.73
SCODI6	4.52	0.91	−2.43	6.54
SCODI7	4.24	1.37	−1.59	0.95
SCODI8	3.81	1.25	−0.77	−0.49
SCODI9	4.42	0.88	−1.64	2.34
SCODI10	4.70	0.83	−3.18	9.87
SCODI11	4.77	0.64	−3.19	10.73
SCODI12	4.79	0.58	−3.49	14.13
SCODI13	4.46	1.09	−2.06	3.21
SCODI14	3.80	1.26	−0.82	−0.36
SCODI15	3.38	1.38	−0.34	−1.01
SCODI16	3.64	1.69	−0.69	−1.30
SCODI17	3.54	1.36	−0.57	−0.76
SCODI18	4.35	1.10	−1.85	2.75
SCODI19	3.58	1.66	−1.08	−0.07
SCODI20	3.68	1.71	−1.14	−0.05
SCODI21	4.15	1.28	−1.41	0.77
SCODI22	3.38	1.44	−0.47	−1.13
SCODI23	2.09	1.43	0.97	−0.54
SCODI24	4.13	1.40	−1.50	0.87
SCODI25	3.94	1.29	−1.06	0.02
SCODI26	3.38	1.37	−0.33	−1.11
SCODI27	4.13	1.23	−1.38	0.81
SCODI28	2.17	1.47	0.83	−0.77
SCODI29	3.74	1.94	−1.24	−0.23
SCODI30	3.82	1.07	−0.99	1.06
SCODI31	3.81	1.10	−0.78	0.01
SCODI32	4.55	0.84	−2.62	8.35
SCODI33	4.25	1.03	−1.58	2.10
SCODI34	4.34	1.05	−1.85	2.85
SCODI35	4.43	1.01	−1.98	3.38
SCODI36	4.09	1.31	−1.47	1.28
SCODI37	4.22	1.14	−1.48	1.31
SCODI38	4.28	1.04	−1.73	2.66
SCODI39	4.11	1.19	−1.52	1.83
SCODI40	4.11	1.18	−1.35	0.96

SCODI items from Ausili et al. (20).

Although respondents were more likely to have problems regulating self-care management (62.85 opinions; SD = 20.87), they also expressed high confidence in self-care (79.56 opinions, SD = 21.34). Table 3 presents the characteristics of the 4 SCODI subscales used in the Spanish version of the inventory.

**Table 3:** Characteristics of the SCODI subscales.

SCODI	*N*	Mean	SD	Median	Lo	Hi	Range
Self-care maintenance items 1–12	218	78.24	14.59	81.25	8.33	100	91.67
Self-care monitoring items 13–20	218	71.81	21.34	76.47	5.88	100	94.12
Self-care management items 21–29	218	62.85	20.87	66.66	0	100	100
Self-care confidence items 30–40	218	79.56	21.34	86.36	0	100	100

The following are abbreviations: *N*-number of participants; SD-standard deviation; Lo-lowest value; Hi-highest; SCODI-Self-Care of Diabetes Inventory.

A factor analysis was performed by the principal component’s method with Varimax rotation. A KMO = 0.82 was obtained and Bartlet’s test of spheridicity was significant (*p* < 0.0001), fitting the model.

Cronbach’s alpha and discriminant powers. Cronbach’s alpha for the 4 subscales were as follows: self-care confidence = 0.936; self-care maintenance = 0.766; self-care monitoring = 0.790; and self-care management = 0.771. Table 4 shows the individual reliability of the SCODI questionnaire items. It is not necessary to change the scales of any of the questions because, as can be seen from the table, they contain positive discriminant powers (item-total correlations).

**Table 4:** SCODI questionnaire reliability analysis.

Scale	Item	Cronbach’s Alpha item removal	Discriminating power (item-total correlation)
Self-care maintenance	1	0.741	0.479
	2	0.772	0.318
	3	0.742	0.495
	4	0.742	0.471
	5	0.78	0.144
	6	0.749	0.428
	7	0.777	0.216
	8	0.736	0.519
	9	0.732	0.619
	10	0.737	0.586
	11	0.743	0.579
	12	0.751	0.482
Self-care monitoring	13	0.756	0.606
	14	0.767	0.5
	15	0.79	0.344
	16	0.764	0.524
	17	0.765	0.509
	18	0.758	0.593
	19	0.757	0.557
	20	0.778	0.448
Self-care management	21	0.731	0.593
	22	0.731	0.572
	23	0.759	0.383
	24	0.732	0.566
	25	0.732	0.581
	26	0.751	0.438
	27	0.732	0.592
	28	0.791	0.166
	29	0.778	0.332
Self-care confidence	30	0.931	0.713
	31	0.932	0.69
	32	0.93	0.745
	33	0.928	0.77
	34	0.93	0.726
	35	0.93	0.728
	36	0.94	0.542
	37	0.927	0.785
	38	0.925	0.847
	39	0.93	0.737
	40	0.926	0.819

Items from Ausili et al. (20).

Confirmation Factor Analysis (CFA). Taking the structure of the Spanish version of SCODI, both CFI (confirmatory fit index), TLI (Tucker-Lewis index), SRMR (standardized root mean square residual) and RMSEA (root mean square error of approximation) yielded adequate results for this composition. This enabled us to verify the 4-component tool’s original makeup.

Table 5 present the findings.

**Table 5:** CFA fit indices of tested SCODI models.

			95% Confidence intervals		
Type	SRMR	RMSEA	Lower	Upper	RMSEA *p*
Robust	0.086	0.039	0.032	0.045	0.999

A CFI value of ≥0.95 indicates a good model fit, while values between 0.90 and 0.95 suggest a satisfactory fit. This measure assesses how much the proposed model improves the fit compared to a null model, with higher values indicating better fit to the data. Similarly, an SRMR close to <0.08 is considered a good fit, while values between 0.08 and 0.10 are seen as acceptable; it represents the average difference between observed correlations and model predictions, with lower values indicating a more accurate fit. Regarding the RMSEA, a value of <0.06 indicates a strong fit, and values between 0.06 and 0.08 are considered acceptable; this measure takes into account both sample size and model complexity, and a low RMSEA suggests a good fit of the model to the data. Furthermore, the model’s chi-square index yielded a score of 1.028 with 773 degrees of freedom.

**Figure 1:**
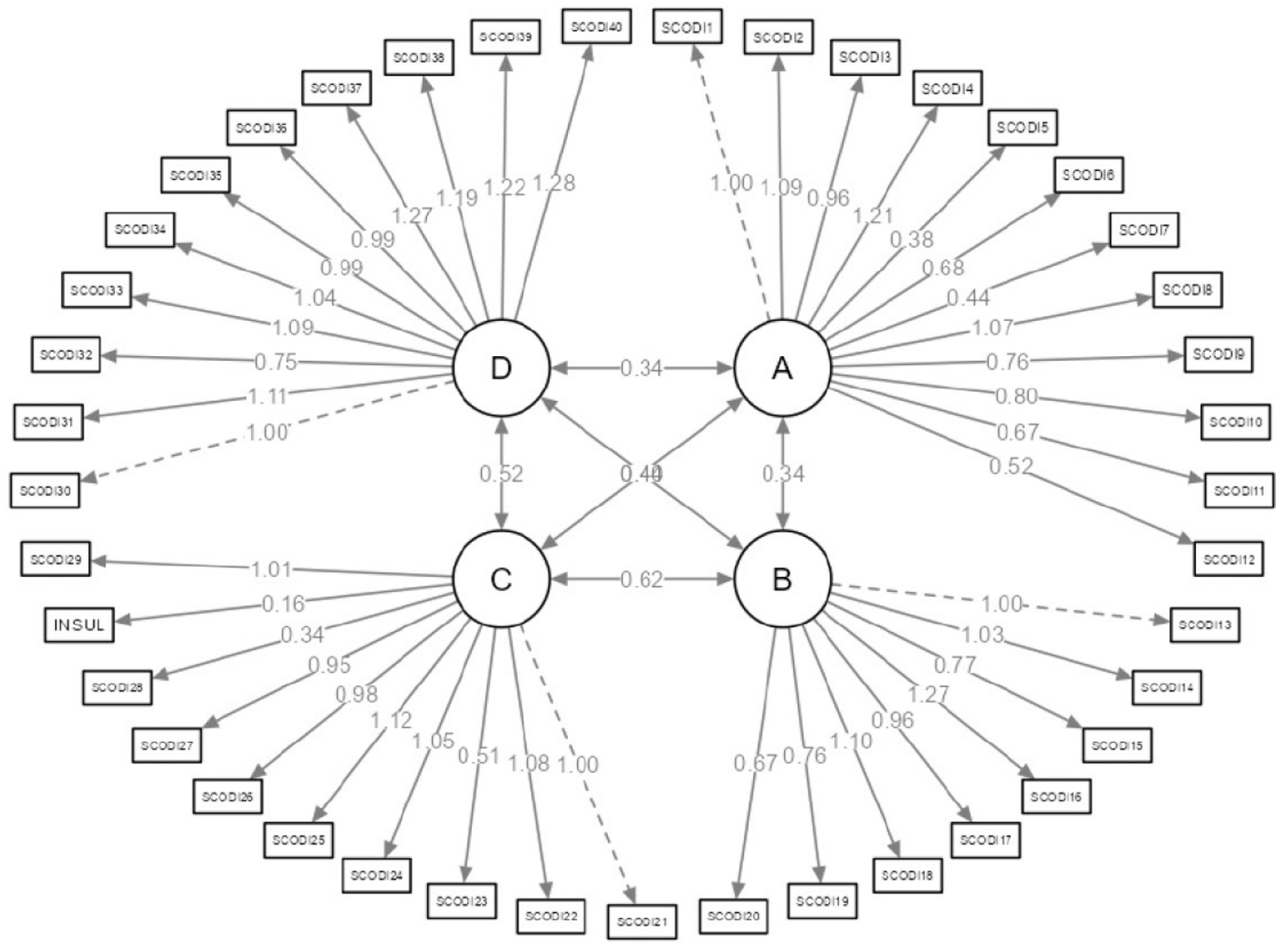
Confirmatory analysis for the Spanish version of the SCODI.

Figure 1 illustrates the confirmatory analysis of the Spanish version of the SCODI, displaying non-standardized factor loadings. The latent variables A, B, C, and D are utilized to represent theoretical constructs that cannot be directly measured but are instead inferred from other observable variables. The rectangles symbolize the questions of the SCODI, which act as the measurements for evaluating the latent variables. The arrows pointing from the latent variables to the observed variables indicate that the latent variables influence the observed variables. The numbers accompanying the arrows represent the regression coefficients (loading factors), which signify the strength and direction of the relationship.

## 4 Discussion

The aim of this study was to obtain a valid and reliable Spanish version of the SCODI after translating and culturally adapting the original version. To do so, it was necessary to test the psychometric properties of the new tool.

The original SCODI questionnaire was developed on the basis of the mid-range theory of chronic diseases, and measures maintenance, monitoring, management and confidence in self-care (20). Our sample of 218 people with T1DM and T2DM answered the Spanish version of the SCODI and demonstrated that it is a valid and reliable tool for measuring self-care. The SCODI’s original structure consists of four factors. The structure of the translated and adapted Spanish versions showed satisfactory values for the SRMR, RMSEA, CFI, and TLI fit indices. Therefore, we confirm that the Spanish translation and adaptation of this composition were as successful as the Italian, American or Polish versions (10, 20, 22). Cronbach’s alpha was used to determine the internal consistency of the Spanish inventory. Several studies have proposed the following classifications for the internal consistency of the items: scores ≥0.9 = excellent; ≥0.8 = good; ≥0.7 = acceptable; ≥0.6 = dubious; ≥0. 5 = deficient; and <0.5 as inadmissible. Nevertheless, the coefficient truly has no lower bound (27). The Cronbach’s alpha for the specific scales in our sample of 218 patients was 0.766 for maintenance of self-care, 0.790 for monitoring self-care, 0.771 for management self-care, and 0.936 for confidence in self-care. The aforementioned values resemble those recorded in the initial SCODI version (respectively: 0.81, 0.84, 0.86, 0.89) (20), the Polish version (respectively: 0.759; 0.741; 0.695; 0.932) (22), the Korean version (respectively: 0.777; 0.69; 0.81; 0.90) (21), or the Farsi version (respectively: 0.81; 0.76; 0. 59; 0.88) (23). We used the chi-square index divided by the degrees of freedom because the chi-square depends on the sample size. As the result obtained is less than 3, it can be stated that the model fit is correct. The psychometric properties of the Spanish version of the SCODI questionnaire are strong, likened to the original versions and other international adaptations. Notably, it excels in high internal consistency in the self-care confidence scale and exhibits satisfactory model fit indices. Consequently, we can deduce that the Spanish version matches the validity and reliability of the Italian, American, and Polish versions, and in certain aspects, even surpasses the Korean and Farsi versions.

Research and clinical practice can make use of the SCODI. It provides healthcare professionals with insights and understanding of the characteristics of people with DM and their strengths and weaknesses with respect to self-care in a quick, simple and reliable way. In this way, interventions can be personalized, forming groups to work on the skills or knowledge identified as deficient for effective self-care. SCODI can therefore be considered a tool that helps the sustainability and efficiency of the healthcare system.

To facilitate comparisons, all scale and subscale scores must be standardized in the range of 0–100. Similar to the authors of the tool, we advise against calculating an overall SCODI score. A score equal to or greater than 70 points on any of the 4 subscales indicates adequate self-care in that component (6, 20). The mean scores of our sample, for the standardized scores obtained in the four SCODI scales, show adequate behaviors in maintenance, monitoring, and confidence, and inadequate behaviors in management. These findings are consistent with those of previous international studies (23, 43), and although they should be followed up in subsequent studies, they highlight the need to intensify efforts to increase knowledge about both consultative and autonomous self-care management behaviors in people with DM.

Given the economic challenges faced by healthcare systems worldwide and the increasing prevalence of DM globally, prioritizing self-care has become imperative as a primary strategy for achieving optimal disease management. Taking into account the fundamental principle of self-care in diabetes, care should be patient-centered. People with chronic diseases should take responsibility for their health and be actively involved in self-care (42). Patients spend an average of 66 min per year with health experts (44). The rest of the time, it should be people with chronic conditions, such as DM, and their caregivers who are responsible for health maintenance, disease prevention, monitoring and disease management; these are known as self-care activities (45). Supporting and empowering individuals with DM and their caregivers and helping them protect themselves can improve patients’ well-being, reduce morbidity and mortality, and minimize healthcare prices (46). However, scholars have highlighted the difficulty of self-care and the vast diversity of components that influence individuals’ self-care choices. These factors can be grouped into behavioral changes and disease-related factors (42). Financial difficulties (47), the effects of emotions or moods (48, 49), personality traits (50) or social factors (51) are some of these factors. To improve patient outcomes by implementing effective and efficient self-care interventions, a dedicated research program is required to deepen the theoretical understanding of self-care criteria and mechanisms underlying self-care behaviors. The efficacy of self-care interventions will remain uncertain and unsatisfactory unless there is a deeper understanding of when, why, and how these interventions work or fail (42).

In the modern world, information sharing is crucial for advancement in all scientific fields. As a consequence, assessment instruments developed in one region are frequently applied in another, generating the need for them to be compatible and therefore culturally adapted to each country. The World Health Organization (WHO) recommends the development and use of standardized health indicators. In particular, it requires international multicenter research to have validated instruments in the field of health in order to be able to carry out international comparative studies (52). Each region has unique cultural traits, beliefs, customs and social behaviors, both nationally and within specific groups. In other words, the same is true in self-care activities as in other facets of life. Although the fundamentals of self-care, such as eating a balanced diet and exercising, are universal, the details vary. The Spanish healthcare system, which is free and universal, belongs to the group of systems in which individuals may perceive health and healthcare products and services as a “right,” which may unintentionally lead to a phenomenon of dependence on nursing care and medical treatment, discouraging self-care (42). Different factors such as the economic crisis, advanced technology and high costs of health procedures, or the increase in life expectancy, have provoked symptoms of exhaustion of the health system itself (53). The empowerment of diabetics regarding their self-care is one of the key tactics in system resilience (54, 55). To this end, SCODI efficiently identifies those specific points of maintenance, monitoring, management or confidence in self-care in which individuals present weaknesses, posing a danger of ineffective self-care (20). This detection is crucial because it signals the potential onset of complications and an increase in comorbidities. Moreover, SCODI allows the identification of those areas or factors of diabetes self-care where the person does not require intervention, thereby saving unnecessary resources and effort. Owing to the complexity and multifactorial nature of the issue, it is critical to step up efforts to identify those who require additional support and are unable to continue with the recommendations. In this way, healthcare teams can design personalized programs and interventions for health education, monitoring techniques, screening for complications, self-help groups, personal confidence and motivation, or individually adjust the frequency of consultations with healthcare professionals. SCODI allows us to reliably measure four key aspects of self-care in diabetics (maintenance, monitoring, management and confidence in self-care). Thanks to this, it has been possible to study and improve knowledge of the behavior of the four dimensions of self-care when influenced by caregivers (56), the influence of different sociodemographic and clinical determinants (6), the influence of adherence to treatment (57), how self-care influences a value like HbA1c (58), the impact of cognitive impairment on self-care (59), analyze the impact of disease acceptance, demographic and clinical variables on adherence to self-care recommendations (60), how the scientific language of self-care is used (13), assess the quality of life of different groups of people with DM (61), investigating the effects of a self-care promotion program on self-care behaviors (62), compare self-care in different groups of people with DM (9) and even from different cultures (10). The options for increasing knowledge about the different variables affecting self-care have increased since the development of SCODI. Aspects such as the influence of anxiety, depression or rest, or the impact of different educational or health programs on the self-care of people with DM should be studied in the future.

Studies showing how self-care can regulate the pathology in persons with DM are still lacking in Spain; this is likely because there is no validated tool to measure the various aspects, which generates a lost opportunity phenomenon. The studies carried out refer to the evaluation of specific variables that intervene in the pathology, such as self-control of glycemia (63), but they do not do so in a global and complete manner like SCODI (64, 65). Translation and cultural adaptation of this questionnaire also allow comparison of the efficacy of different specific social and health interventions. Continuous glucose monitoring devices should be offered to people with DM and health professionals should acquire management, promotion and training skills (66). Continuous glucose monitoring is also accepted for the work of researchers (67). SCODI allows us to monitor the relationship between these devices and self-care. As an example, our study, in which the sample is obtained in a territory where flash glucose monitors in people with T1DM are 100% funded by the public health system, i.e., they are free. This factor is reflected in the result of SCODI item 13 (Do you regularly monitor your blood sugar levels?) for the group of persons with T1DM (Mean: 4.92; SD: 0.32). Comparison of this result with those of a sample from another Spanish territory with paid glucose flash monitors would illustrate the efficacy of the program. We cannot do so with the results of patients with T2DM in our sample (Mean: 3.96; SD: 1.34) because it has started to be free of charge during the data collection process also for these individuals.

There are limitations in our study, starting with convenience sampling, which may not be sufficiently representative, limiting the scope of our investigation. However, all SCODI translation and validation work published to date has used the same method. The participants were recruited from a hospital surgical ward; the reason for admission was mostly not directly related to diabetes. Only 9.3% were hospitalized for that reason in the last 12 months, and most sought diabetes care through health centers and outpatient clinics (Table 1). This may ameliorate the bias of convenience sampling. In our research, 14.12% of the selected individuals agreed to participate, and the rest refused to participate. The surgical origin of the admission made it difficult to motivate participation in the study, but the choice of the subjects was respected at all times, and we did not observe that this could represent a selection bias. Second, the cross-sectional character of the analysis is a restriction; hence, it is possible that longitudinal construct validity reviews may need to be performed in future research. All SCODI validation studies employed cross-sectional designs. In addition, reported self-care may not accurately represent actual self-care practices. Ultimately, the sample size (218) could be increased, although it is sufficient to assess the main objectives of the analysis. However, it should be noted that only the Farsi (400) (23) and Polish (276) (22) versions have a larger sample size. The studies of Korean (210) (21), Italian (200) (20), and English (226) (10) versions are similar.

## 5 Conclusion

Our analysis confirms the validity, reliability, and reproducibility of the tool used to assess self-care in patients with T1DM and T2DM within the Spanish context. The extensive literature on the original SCODI has thoroughly demonstrated its construct validity, reliability, and content validity, as well as the generalizability of its measurement model. Publications validating versions in Farsi, Polish, and Korean, alongside the already established Italian and English versions, consistently affirm their satisfactory validity and reliability.

The development of a Spanish version holds strategic importance for international comparisons focusing on maintenance, follow-up, management, and confidence in self-care. Such cross-cultural assessments can be correlated with sociodemographic, economic, or health factors, contributing significantly to the formulation of global strategies for diabetes management by both clinicians and researchers. There are challenges in the self-care of people with DM, such as behavioral changes, disease-related factors or the influence of culture on behavioral choices that the Spanish version of SCODI will help to address. SCODI serves as a valuable tool for predicting the risk of complications stemming from ineffective self-care across its dimensions, enabling the implementation of preventive measures that enhance overall health and optimize healthcare expenditure.

## 6 Limitations

Our study hones in on examining the internal validity of the questionnaire, with the goal of guaranteeing its internal consistency and describing its psychometric properties. The SCODI is a widely utilized tool, and the translation process ensures its content validity. Employing EFA-CFA on the identical sample effectively aligns with the questionnaire’s primary objectives of enhancing its psychometric properties.

## Data availability statement

The raw data supporting the conclusions of this article will be made available by the authors, without undue reservation.

## Ethics statement

The studies involving humans were approved by the Ethics Committee of the University of La Rioja (reference number CEImLAR P.I. 572). The studies were conducted in accordance with the local legislation and institutional requirements. The participants provided their written informed consent to participate in this study.

## Author contributions

JM-T: Conceptualization, Data curation, Formal analysis, Funding acquisition, Investigation, Methodology, Project administration, Resources, Software, Supervision, Validation, Visualization, Writing – original draft, Writing – review & editing. DA: Software, Supervision, Validation, Visualization, Writing – original draft. NS-R: Formal analysis, Project administration, Supervision, Validation, Writing – original draft. IS-A: Formal analysis, Methodology, Supervision, Validation, Writing – original draft. AD: Investigation, Methodology, Project administration, Resources, Writing – original draft. MN: Conceptualization, Methodology, Software, Validation, Visualization, Writing – original draft. YL: Funding acquisition, Methodology, Resources, Validation, Visualization, Writing – original draft. RV-H: Methodology, Project administration, Supervision, Validation, Visualization, Writing – original draft. CT-G: Conceptualization, Data curation, Formal analysis, Investigation, Methodology, Software, Writing – original draft. MS: Formal analysis, Methodology, Writing – original draft. VG-C: Formal analysis, Investigation, Methodology, Project administration, Supervision, Validation, Writing – original draft, RJ-V: Data curation, Funding acquisition, Methodology, Resources, Supervision, Visualization, Writing – original draft.

## Footnotes

^1^ www.self-care-measures.com

^2^ https://self-care-measures.com/self-care-scoring-algorithm/
